# Exploring the Roles of Dispositional Mindfulness and Cognitive Reappraisal in the Relationship Between Neuroticism and Depression Among Postgraduate Students in China

**DOI:** 10.3389/ijph.2022.1605074

**Published:** 2022-08-25

**Authors:** Lulu Hou, Lei Chang, Lirong Chen, Junfeng Fei, Renlai Zhou

**Affiliations:** ^1^ Department of Psychology, Nanjing University, Nanjing, China; ^2^ Department of Psychology, Shanghai Normal University, Shanghai, China; ^3^ Department of Psychology, Faculty of Social Sciences, University of Macau, Macau, China; ^4^Department of Psychology, Suzhou University of Science and Technology, Suzhou, China; ^5^ Institute of Education, Nanjing University, Nanjing, China; ^6^ State Key Laboratory of Media Convergence and Communication, Communication University of China, Beijing, China

**Keywords:** depression, neuroticism, dispositional mindfulness, cognitive reappraisal, moderated mediation model

## Abstract

**Objectives:** Previous studies revealed a positive association between neuroticism and depression. This study further extended the previous findings by exploring the psychological processes underlying this association among Chinese postgraduates. Guided by theoretical models and empirical research, we proposed a multiple mediation and moderated mediation model to investigate the roles of dispositional mindfulness and cognitive reappraisal in the relationship between neuroticism and depression.

**Methods:** Using the NEO Five-Factor Inventory, Beck Depression Inventory, Mindfulness Attention Awareness Scale, and Emotion Regulation Questionnaire, 1103 first-year postgraduates at a comprehensive university in China were surveyed. Path analysis was adopted to test the models.

**Results:** The results showed that dispositional mindfulness mediated the association between neuroticism and depression. Further, this mediating effect was moderated by cognitive reappraisal, with this effect being stronger in individuals with low engagement in cognitive reappraisal.

**Conclusion:** The results support interrelations among neuroticism, depression, dispositional mindfulness, and cognitive reappraisal as moderated mediation rather than multiple mediation. The results enhance our understanding of psychological mechanisms between neuroticism and depression and provide suggestions for interventions to prevent or reduce depression in highly neurotic postgraduates.

## Introduction

On 23 February 2017, the World Health Organization reported that 322 million people suffer from depression worldwide. Among them, young people, pregnant and postpartum women, and elderly are especially vulnerable to depression [1]. Evans et al. (2018) evaluated 2279 postgraduates who completed clinically validated scales for anxiety and depression via social media and email, and reported that 41% of postgraduates manifested moderate-to-severe anxiety and 39% of postgraduates exhibited moderate-to-severe depression [2]. Similar mental health issues were prevalent in China before [3] and during [4] the Coronavirus disease (COVID-19) pandemic. Gewin (2012) stated that depression was prevalent among postgraduate and postdoctoral students, who experienced pressure from academic studies, interpersonal relationships, and career planning, even in the first year of study [5, 6]. Accordingly, it is crucial to investigate and prevent depression in postgraduates.

The five-factor model of personality is one of the most influential personality theories [7]. The five factors, labeled extraversion, neuroticism, openness, agreeableness and conscientiousness, were identified in self-rating and peer rating scales [8, 9], and in populations with personality disorder [10]. Among the five factors, neuroticism indicates emotional instability and predicts negative outcomes in daily life, and is a powerful predictor of depression, anxiety, and other emotional disorders [11–13]. Although extant findings suggest the compelling role of neuroticism in the etiology and maintenance of depression, little is known about these associations among postgraduate students, and the factors underlying this relationship are yet to be explored. Therefore, this study further extended previous studies by exploring the psychological processes underlying the association between neuroticism and depression and examining the roles of dispositional mindfulness and cognitive reappraisal among Chinese postgraduates.

### Mediation Role of Dispositional Mindfulness

Mindfulness is defined as the state of being attentive to and aware of current situations [14]. Accordingly, dispositional mindfulness refers to an individual’s ability to be attentive to and aware of the present situation, i.e., self-awareness [15]. However, individuals with severe neuroticism fear of potential future threats (i.e., worry) and reflect more on previous negative emotional experiences (i.e., rumination) than individuals with less severe neuroticism [16, 17]. Thus, individuals with severe neuroticism face difficulty in concentrating their attention and consciousness on their present feelings in addition to problems entering a state of mindfulness [18, 19]. Accordingly, their levels of dispositional mindfulness tend to be low.

Previous studies also revealed negative relationships between dispositional mindfulness and depression in specific populations and occupations [20, 21] as well as general population during COVID-19 pandemic [22, 23]. This may be because attentiveness and awareness of the present situation help individuals disengage from previous negative emotional experiences, as claimed by Desrosiers et al. (2013) [24]. In addition, Giluk (2009) conducted a meta-analysis and revealed strong negative correlations between dispositional mindfulness and neuroticism (*r* = −0.45) and between dispositional mindfulness and trait negative affect (*r* = −0.39) [25]. Thus, we hypothesized that dispositional mindfulness mediates the relationship between neuroticism and depression.

It should be noted that although neuroticism and dispositional mindfulness are both trait types, they are distinct at the hierarchical level from the perspective of core versus surface characteristics [26]. Neuroticism is considered a core characteristic, a pattern of thoughts, feelings, and actions that is not susceptible to change over time or by the situation. In contrast, dispositional mindfulness is considered a surface characteristic, that is, a trait that emerges later and is less stable than the core traits and can be cultivated and strengthened through various interventions, such as training programs [27]. Empirical research evidence seems to be consistent with this view. Neuroticism is associated with being more genetically based, whereas dispositional mindfulness has been found to be more influenced by the environment, such as attachment styles, child abuse, organizational climate of care, and trauma exposure [28–32]. This is also supported by evidence of heritability, which ranges from 40% to 60% for neuroticism [33], but only 32% for dispositional mindfulness [34]. Thus, it appears that dispositional mindfulness is a less stable and more environmentally susceptible surface manifestation of personality. Thus, the relationship between neuroticism and dispositional mindfulness reflects the interaction of different levels of traits, meaning that dispositional mindfulness is similar to the interaction between neuroticism and environmental factors. This may also be the reason why many researchers are interested in the relationship between personality factors and dispositional mindfulness [25, 35–37].

### Role of Cognitive Reappraisal

Cognitive reappraisal refers to thinking about events in an objective, neutral, and positive manner [38]. A greater tendency to engage in cognitive reappraisal is associated with fewer symptoms of depression [39]. Further, high levels of neuroticism are associated with a lower tendency to engage in cognitive reappraisal [40]. Therefore, individuals with less severe neuroticism may be less likely to experience depression because they tend to engage in cognitive reappraisal and thus more effectively regulate negative emotion.

Further, focusing on the present may expand the awareness of various stimuli, so as to identify negative cognition and emotions more accurately and promptly [41]. Previous studies suggested that high dispositional mindfulness was associated with high consistency between implicit and explicit measures of emotion [14], which indicated that individuals with high dispositional mindfulness are more aware of their feelings. In addition, enhanced awareness increases the information available to individuals, which facilitates successful reappraisal of the current situation. Empirical studies found that mindfulness training [42] especially state mindfulness during meditation [43] was associated with an increased tendency to engage in cognitive reappraisal. Accordingly, we hypothesized that cognitive reappraisal mediates the relationships between neuroticism and depression and between dispositional mindfulness and depression.

However, developing reappraisal skills is not a specific goal of mindfulness practices [24]. Thus, the association between dispositional mindfulness and depression possibly depends on the frequency of engagement in cognitive reappraisal. Although most previous studies have assessed the role of cognitive reappraisal as a mediator in the relationship between dispositional mindfulness and outcomes [44, 45], it is also reasonable to assume that individuals with high levels of dispositional mindfulness may be less susceptible to depression for those able to use cognitive reappraisal easily than those who use cognitive reappraisal hard and the ease of using cognitive reappraisal depends on the recency and frequency of strategies used in similar emotional situations [46]. Accordingly, cognitive reappraisal plays a moderated mediation role in the relationship between neuroticism and depression.

### Current Study: Testing Moderated Mediation and Multiple Mediation Models

**FIGURE 1:**
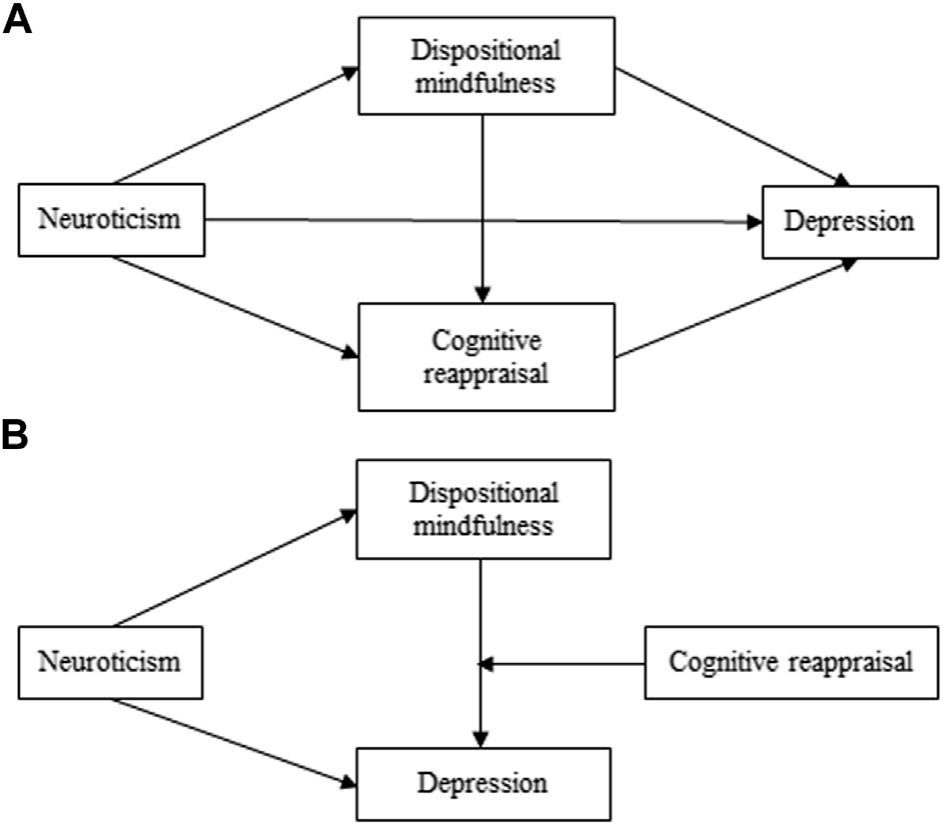
Conceptual model of the effect of neuroticism on depression (China, 2017). **(A)** The multiple mediation model. **(B)** The moderated mediation model.

This study first assessed whether dispositional mindfulness mediated the relationship between neuroticism and depression among first-year postgraduates in China. Further, this study examined the role of cognitive reappraisal in the relationship between neuroticism and depression. Based on a literature review, this study proposed the following three hypotheses. Support for H1 and H2 suggests a multiple mediation relationship (see Figure 1A), whereas support for H1 and H3 suggests a moderated mediation relationship (see Figure 1B).

H1 Dispositional mindfulness mediates the relationship between neuroticism and depression.

H2 Cognitive reappraisal mediates the relationship between neuroticism and depression. In addition, neuroticism negatively predicts dispositional mindfulness, which predicts more frequent cognitive reappraisal and thereby lower levels of depression.

H3 Cognitive reappraisal moderates the mediating effect of dispositional mindfulness on the relationship between neuroticism and depression. This mediating effect is stronger in individuals with low engagement in cognitive reappraisal.

## Methods

### Participants

We used convenience sampling to recruit 1200 newly enrolled postgraduates at a comprehensive university in China. We excluded invalid responses with incomplete answers (constituting at least 20% of the unanswered questions, and at least one blank questionnaire), inconsistent answers (with clear contradiction), or obvious regularities (e.g., choosing one answer for at least 10 consecutive questions). Accordingly, 1103 students (55.30% female) completed the survey with an effective response rate of 91.92%. The average age of the participants was 24.32 ± 2.92 years, ranging from 18 to 43 years. Among them, 198 were general doctoral students (including those admitted through general admission and the application exam); 60 were direct postgraduates (equivalent to first-year master’s students); 456 were academic master’s students; 351 were professional master’s students; and 32 were business administration master’s students. The degree programs of 6 participants were unspecified.

This study was approved via the Ethical Evaluation of Research Projects at the Department of Psychology in the School for Social and Behavioral Sciences at Nanjing University. All procedures involving human participants were in accordance with the ethical standards of the institutional or national research committee and with the 1964 Declaration of Helsinki and its later amendments or comparable ethical standards. Informed consent was obtained from all participants included in the study.

### Instruments

#### NEO Five-Factor Inventory

The sub-scale of Chinese version of the NFFI was employed to measure participants’ levels of neuroticism [47, 48]. Participants rated 12 items (e.g., “I often feel nervous and uneasy”) from 1 (strongly disagree) to 5 (strongly agree). The scale has an acceptable Cronbach’s alpha score of 0.87.

#### Beck Depression Inventory

The Chinese version of the BDI was employed to measure participants’ depression levels during the preceding week [49, 50]. Participants rated 21 items (e.g., “I am not a loser”) from 0 (no) to 3 (absolutely). The scale has an acceptable Cronbach’s alpha score of 0.83.

#### Mindfulness Attention Awareness Scale

The Chinese version of the MAAS was employed to measure participants’ dispositional mindfulness [14, 51]. Participants rated 15 items (e.g., “I could be experiencing an emotion and not be conscious of it until sometime later”) on a scale of 1 (almost always) to 6 (almost never). The scale has an acceptable Cronbach’s alpha score of 0.87.

#### Emotion Regulation Questionnaire

The Chinese version of the subdimension of cognitive reappraisal of the ERQ was employed to measure participants’ engagement of cognitive reappraisal [52, 53]. Participants rated 6 items (e.g., “When I want to feel fewer negative emotions, I change the way I am thinking about the situation”) on a scale of 1 (strongly disagree) to 7 (strongly agree). The scale has an acceptable Cronbach’s alpha score of 0.81.

### Data Analysis

We used SPSS 22.0 to conduct descriptive statistics and perform correlation analysis. Subsequently, we used the SPSS PROCESS macro to analyze the multiple mediation model (Model 6) and moderated mediation model (Model 14) [54]. Notably, age, gender, and student types were considered covariates in the multiple mediation and moderated mediation models. In addition, the difference between the results of unadjusted and adjusted analysis was minor. The unadjusted analysis results are reported in the Supplementary Materials.

## Results

### Preliminary Analysis

Table 1 presents the descriptive statistics and Pearson correlations of all variables. Neuroticism was positively correlated with depression and negatively correlated with dispositional mindfulness and cognitive reappraisal. Depression was negatively correlated with dispositional mindfulness and cognitive reappraisal. Dispositional mindfulness was positively correlated with cognitive reappraisal.

**TABLE 1:** Descriptive statistics and correlations between all variables (*n* = 1103) (China, 2017).

	1	2	3	4	*M* ± *SD*
1. Neuroticism	1				31.08 ± 8.25
2. Depression	0.64***	1			5.72 ± 5.90
3. Dispositional mindfulness	−0.51***	−0.43***	1		64.24 ± 11.14
4. Cognitive reappraisal	−0.29***	−0.24***	0.34***	1	33.00 ± 5.21

Note: ***p < 0.001.

The average BDI score of the surveyed first-year postgraduates indicated mild depression. The median score (4.0) indicated minimal depression. In addition, a positive rate of depression was calculated. Among all the first-year postgraduates, 596 (54.03%) manifested minimal depressive symptoms (score ≤4), 375 (34.00%) had mild depression (5–13), 99 (8.98%) had moderate depression (14–20), and 33 (2.99%) had severe depression (≥21).

### Testing the Multiple Mediation Model

The main results revealed that after the inclusion of covariates, neuroticism positively predicted depression (*β* = 0.58, *t* = 21.48, *p* < 0.001) and negatively predicted dispositional mindfulness (*β* = − 0.51, *t* = −19.29, *p* < 0.001) and cognitive reappraisal (*β* = − 0.18, *t* = −5.45, *p* < 0.001). Further, dispositional mindfulness negatively predicted depression (*β* = − 0.13, *t* = −4.80, *p* < 0.001) and positively predicted cognitive reappraisal (*β* = 0.26, *t* = 7.91, *p* < 0.001). However, the effect of cognitive reappraisal on depression was not significant (*β* = − 0.02, *t* = −0.86, *p* = 0.39). Accordingly, the multiple mediation model was not supported because of the nonsignificant effect of cognitive reappraisal on depression.

### Testing the Moderated Mediation Model

**FIGURE 2:**
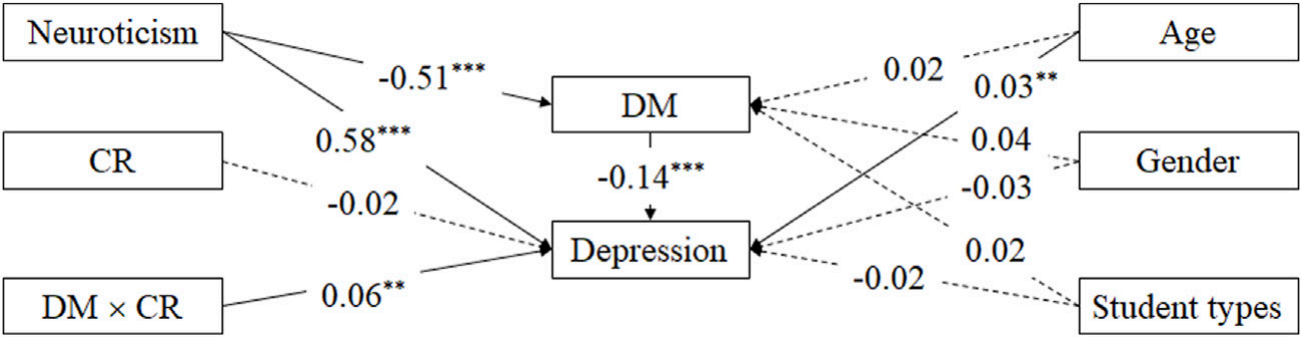
The moderated mediation model of the relationship between neuroticism, dispositional mindfulness, cognitive reappraisal, and depression (China, 2017).

Table 2 and Figure 2 presented the main results generated by the SPSS PROCESS macro (Model 14) [54]. The model comprised a (1) mediator and dependent variable model and (2) conditional indirect effect. The mediator and dependent variable model revealed that after the inclusion of covariates, neuroticism positively predicted depression (*β* = 0.58, *t* = 21.53, *p* < 0.001) and negatively predicted dispositional mindfulness (*β* = − 0.51, *t* = −19.29, *p* < 0.001). Further, dispositional mindfulness negatively predicted depression (*β* = − 0.14, *t* = −5.07, *p* < 0.001). A bootstrap procedure was conducted to assess the size of the indirect effect and obtain confidence intervals (CIs). Using random sampling, we generated 1000 bootstrapping samples from the original dataset. The indirect effect of neuroticism on depression through dispositional mindfulness was 0.07 (95% CI = [0.04, 0.10]). Because the 95% CI did not include 0, dispositional mindfulness was considered to mediate the effect of neuroticism on depression.

**TABLE 2:** The moderated mediation effect of dispositional mindfulness and cognitive reappraisal on neuroticism and depression (China, 2017).

Predictors	Model 1 (dispositional mindfulness)			Model 2 (depression)		
	*β*	*t*	[LLCI ULCI]	*β*	*t*	[LLCI ULCI]
age	0.02	1.66	[−0.00 0.04]	0.03	3.38**	[0.01 0.05]
gender	0.04	0.81	[−0.06 0.15]	−0.03	−0.67	[−0.12 0.06]
student types	0.02	0.75	[−0.03 0.07]	−0.02	−0.93	[−0.06 0.02]
neuroticism	−0.51	−19.29***	[−0.56–0.46]	0.58	21.53***	[0.53 0.63]
DM				−0.14	−5.07^***^	[−0.19–0.08]
CR				−0.02	−0.71	[−0.07 0.03]
DM × CR				0.06	2.70^**^	[0.02 0.10]
*R* ^*2*^	0.26			0.44		
*F*	96.32***			121.97***		

Note: ***p < 0.001, **p < 0.01. DM, dispositional mindfulness; CR, Cognitive Reappraisal. Student types include general doctoral students, direct postgraduates, academic master’s students, professional master’s students, and business administration master’s students.

The interaction between dispositional mindfulness and cognitive reappraisal positively predicted depression (*β* = 0.06, *t* = 2.70, *p* = 0.007). These results indicate that cognitive reappraisal moderates the mediating effect of dispositional mindfulness on the relationship between neuroticism and depression.

**FIGURE 3:**
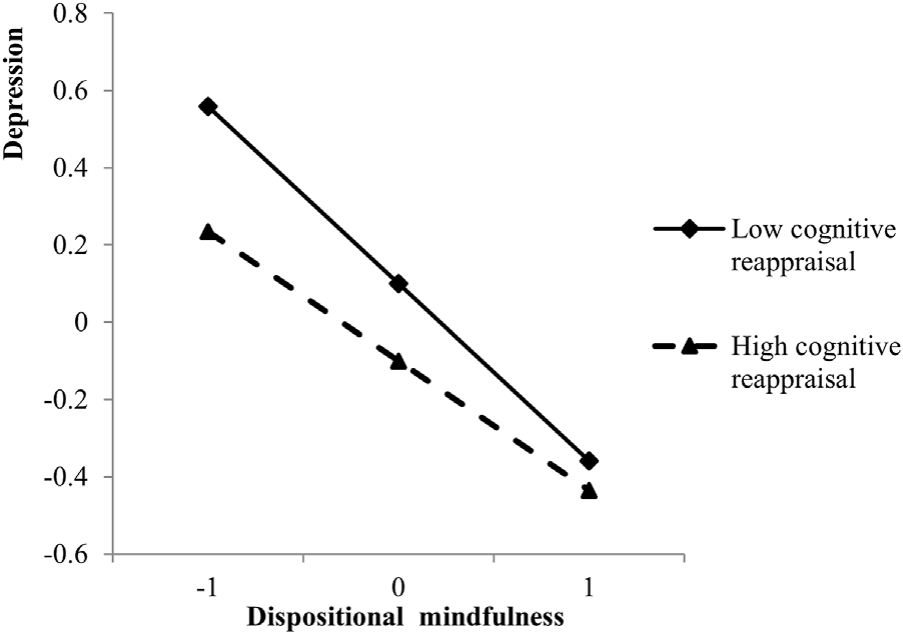
Effect of the interaction between dispositional mindfulness and cognitive reappraisal on depression (China, 2017).

A simple slope test was conducted to further elucidate the moderating effect of cognitive reappraisal. The simple slope test was used to examine the differences in the effect of dispositional mindfulness on depression between individuals who frequently engaged in cognitive reappraisal and those who did not (Figure 3) [55]. For individuals with low cognitive reappraisal scores (mean – 1SD), dispositional mindfulness was significantly negatively associated with depression (*β* = − 0.45, *t* = −9.66, *p* < 0.001). For individuals with high cognitive reappraisal scores (mean + 1SD), this association was significant but weaker (*β* = − 0.34, *t* = −7.86, *p* < 0.001).

## Discussion

This study analyzed the mediating effect of dispositional mindfulness on the relationship between neuroticism and depression. Further, the study determined whether cognitive reappraisal mediates or moderates the aforementioned effect. To our knowledge, this study was the first to investigate the relationship among neuroticism, dispositional mindfulness, cognitive reappraisal, and depression among Chinese postgraduates. The integrated model contributes to our understanding of which reactivity processes link neuroticism and depression and whether these processes differ among individuals. The integrated model also provides empirical evidence of the role of cognitive reappraisal in the relationship among neuroticism, dispositional mindfulness, and depression.

The preliminary analysis revealed that the average BDI scores of the Chinese first-year postgraduates indicated mild depression. Further, nearly half of the participants experienced depression to varying degrees. Although the positive rate differed from the incidence of clinical depression, the findings were consistent with those of an earlier study, which revealed a positive SCL-90 rate of 31.81% among Chinese first-year graduate students [56].

The correlation analysis revealed that neuroticism was positively correlated with depression, which was consistent with those of previous studies involving other age groups [57, 58]. Environment and personality influence emotional response. Although extroversion and neuroticism are both personality traits with typical emotional styles [59], they affect emotional response differently. Specifically, extroversion is related to positive emotional response, whereas neuroticism is related to negative emotional response [60]. When something positive occurs, extroversion triggers strong emotions; a negative event also results in strong emotional behavior, but the emotional state is restored quickly. Neuroticism elicits stronger physiological response and emotional feelings in negative situations [59].

The results of this study elucidate the mediating role of dispositional mindfulness in the relationship between neuroticism and depression. Previous studies have suggested that dispositional mindfulness is correlated with neuroticism and negative emotions [25] and mediated the relationship between neuroticism and subjective well-being or impulsiveness [61]. This study extended previous findings by investigating the role of dispositional mindfulness in mediating the relationship between neuroticism and depression. The results indicate that individuals with severe neuroticism have low dispositional mindfulness, and high depression tendency.

Based on previous results, our study proposed a multiple mediation model including neuroticism, dispositional mindfulness, cognitive reappraisal and depression. Our study suggests that the multiple mediation model was not supported because of the nonsignificant effect of cognitive reappraisal on depression. Notably, the analysis of multiple mediation effects of dispositional mindfulness and cognitive reappraisal revealed significant effects of mediation depending on the strength of the association. The correlation analysis revealed that dispositional mindfulness (−0.51 and −0.43) was more strongly correlated with neuroticism and depression than cognitive reappraisal (−0.29 and −0.24), which may lead to a masking effect as reported previously [62]. Our results indicate that dispositional mindfulness is more strongly associated with psychopathology than cognitive reappraisal. Further, the mediation effect of cognitive reappraisal on the relationship between dispositional mindfulness and depression was not supported, which was inconsistent with the results of previous studies [24]. This inconsistency stems mainly from the role of dispositional mindfulness in the model. For instance, Desrosiers et al. (2013) treated dispositional mindfulness as an independent variable rather than as a mediator as in this study [24].

In addition to the multiple mediation model, our results supported a moderated mediation model considering that cognitive reappraisal skill is not the specific goal of mindfulness practice [24]. Frequency of engagement in cognitive reappraisal strategies influenced the effect of dispositional mindfulness on depression, which may support the orienting attention/action readiness (OAAR) framework used in the emotion regulation field [46]. According to the OAAR framework, the interaction between action readiness and orienting attention determines the outcomes of emotional regulation. Individuals trained in meditation exhibit high levels of dispositional mindfulness and can easily enter a state of orienting attention [63], while individual readiness to implement specific regulatory strategies (e.g., cognitive reappraisal) increases with the frequency of strategies used in similar emotional situations. The present study provides an empirical evidence-based support for the OAAR framework by using attention-oriented ability (i.e., dispositional mindfulness) as a mediating variable and adaptive emotion regulation strategies (i.e., cognitive reappraisal) as a moderating variable. The result indicates that individuals with low dispositional mindfulness have higher depressive tendency; however, the frequency of cognitive reappraisal played a buffering role in this relationship. It is important to note that regardless of the frequency of cognitive reappraisal, with the decrease of dispositional mindfulness, the level of depression increases, but the rate of increase varies with the frequency of using cognitive reappraisal. Thus, cognitive reappraisal only altered the strength of the relationship between dispositional mindfulness and depression, but not the relationship or its direction.

The limitations of this study are notable. First, the research questions were related to causality, but the cross-sectional design and mediation analysis could not indicate causation [64]. Future longitudinal studies are required to specify the directionality of the relationships among neuroticism, dispositional mindfulness, cognitive reappraisal, and depression. Second, we recruited only first-year postgraduates via convenience sampling; thus our data are not representative of all postgraduates. Third, although the interaction between dispositional mindfulness and cognitive reappraisal positively predicted depression, the effect size was relatively small. Caution is therefore essential when drawing conclusions. Fourth, this study explored the roles of dispositional mindfulness and cognitive reappraisal; however, the roles of other emotional regulation strategies (e.g., expressive suppression) remain unexplored. Finally, although neuroticism and dispositional mindfulness are two levels of traits, exploring the relationship between the two traits using subjective scales is less clinically valuable than exploring the relationship between neuroticism and mindfulness using mindfulness-based training. Previous studies found that personality factors (especially the neuroticism dimension) may contribute to the outcome of mindfulness training. For example, de Vibe et al. (2015) found a greater effect of the mindfulness-based intervention on mental distress in highly neurotic students than in lowly neurotic students [65]. Nyklíček1 and Irrmischer (2017) found that highly neurotic participants showed stronger decreases in both anxious and depressed mood than lowly neurotic students [66]. Jagielski et al. (2019) found that the individuals with high levels of neuroticism manifested significantly lower levels of distress at 12-month follow-up compared with those who with low levels of neuroticism [67]. Above all, individuals with high neuroticism tended to be the greatest beneficiaries of mindfulness training. Therefore, we should examine whether baseline neuroticism levels can predict changes in mindfulness and changes in depressive symptoms during mindfulness training to further explore the relationship among neuroticism, mindfulness, and depression in the future.

Despite the aforementioned limitations, this study has key theoretical and practical implications. From a theoretical perspective, this study extends previous research and improves the understanding of how and when individuals with severe neuroticism develop depression. Next, we theoretically clarified the relationship among neuroticism, dispositional mindfulness, cognitive reappraisal and depression using multiple and moderated mediation models with the same sample and variables. Finally, the results provide integrated evidence supporting the OAAR framework. From a practical perspective, our findings are critical for interventions aimed at preventing or reducing depression. Neuroticism has a significant predictive effect on depression. Accordingly, we recommend increased emphasis on mental health education of individuals with severe neuroticism. Further, neuroticism predicted depression not only directly but also through mediation via dispositional mindfulness. Accordingly, individuals with severe neuroticism can be assisted via mindfulness-based interventions. In addition, cognitive reappraisal played a moderating role in the relation between dispositional mindfulness and depression. Thus, interventions combining cognitive reappraisal and mindfulness (e.g., Mindfulness-Based Cognitive Therapy or MBCT) were more effective than mindfulness solely (e.g., Mindfulness-Based Stress Reduction or MBSR) for individuals with severe neuroticism to reduce depression. Querstret et al. (2020) reported a meta-analysis indicating that MBCT generated larger effect sizes than MBSR for depression, suggesting the possibility that the cognitive component of MBCT may contribute to participants’ mindfulness learning, thereby increasing self-reported mindfulness skills [68]. The results of this study using a subjective questionnaire also demonstrated the superiority of MBCT, and therefore MBCT should be used in future clinical practice whenever possible. Of course, a comparative clinical intervention study should be conducted in the future to further validate the differences between the two approaches.
