# Supplementary material for: Exploring the Roles of Dispositional Mindfulness and Cognitive Reappraisal in the Relationship Between Neuroticism and Depression Among Postgraduate Students in China
Source: Int J Public Health. 2022 Aug 25;67:1605074. doi: 10.3389/ijph.2022.1605074 (PMC9452625; doi:10.3389/ijph.2022.1605074)
Supplement: Supplementary file 1 [file DataSheet1.docx]

**International Journal of Public Health**

**Exploring the roles of dispositional mindfulness and cognitive reappraisal in the relationship between neuroticism and depression ﻿among postgraduate students in China**

**Supplementary materials**

**Unadjusted analysis results:**

**Testing for multiple mediation model**

The main results generated by the SPSS PROCESS macro (Model 6) [1] revealed neuroticism positively predicted depression (*β* = 0.57, *t* = 21.17, *p* < 0.001) and negatively predicted dispositional mindfulness (*β* = − 0.51, *t* = −19.63, *p* < 0.001) and cognitive reappraisal (*β* = − 0.16, *t* = −4.96, *p* < 0.001). Furthermore, dispositional mindfulness negatively predicted depression (*β* = − 0.13, *t* = −4.78, *p* < 0.001) and positively predicted cognitive reappraisal (*β* = 0.26, *t* = 7.90, *p* < 0.001). However, the effect of cognitive reappraisal on depression was not significant (*β* = − 0.02, *t* = −0.98, *p* = 0.33). Therefore, the multiple mediation model was not supported due to the effect of cognitive reappraisal on depression was nonsignificant.

**Testing for moderated mediation model**

The main results generated by the SPSS PROCESS macro (Model 14) [1] are presented in **Table S1** and consisted of two parts: (1) a mediator and dependent variable model and (2) moderated indirect effect analysis. As revealed by the mediator and dependent variable model, neuroticism positively predicted depression (*β* = 0.57, *t* = 21.24, *p* < 0.001) and negatively predicted dispositional mindfulness (*β* = − 0.51, *t* = −19.63, *p* < 0.001). Furthermore, dispositional mindfulness negatively predicted depression (*β* = − 0.14, *t* = −5.05, *p* < 0.001). A bootstrap procedure was conducted to assess the size of the indirect effect and confidence intervals (CIs). We generated 1000 bootstrapping samples from the original dataset through random sampling. The indirect effect of neuroticism on depression through dispositional mindfulness was 0.07 (95% CI = [0.04, 0.10]). Based on the empirical 95% CI not including 0, dispositional mindfulness mediated the impact of neuroticism on depression.

Interaction between dispositional mindfulness and cognitive reappraisal positively predicted depression (*β* = 0.06, *t* = 2.71, *p* < 0.01). These results indicate that cognitive reappraisal moderated the mediating effect of dispositional mindfulness on the relationship between neuroticism and depression, and thus moderated mediation model was supported.

**Table S1** The moderated mediation effect of dispositional mindfulness and cognitive reappraisal between neuroticism and depression. ﻿(collected from September 1^st^ to 31^st^, 2017, China)

| Predictors | Model 1 (dispositional mindfulness) | | | Model 2 (depression) | | |
| --- | --- | --- | --- | --- | --- | --- |
|  | *β* | *t* | [LLCI ULCI] | *β* | *t* | [LLCI ULCI] |
| neuroticism | −0.51 | −19.63^***^ | [−0.56 −0.46] | 0.57 | 21.24^***^ | [0.52 0.62] |
| DM |  |  |  | −0.14 | −5.05^***^ | [−0.19 −0.08] |
| CR |  |  |  | −0.02 | −0.81 | [−0.07 0.03] |
| DM × CR |  |  |  | 0.06 | 2.71^**^ | [0.02 0.10] |
| *R^2^* | 0.26 |  |  | 0.43 |  |  |
| *F* | 385.47^***^ |  |  | 207.04^**^ |  |  |

Note: ^***^ *p* < 0.001, ^**^ *p* < 0.01. DM = Dispositional Mindfulness; CR = Cognitive Reappraisal.

**Reference:**

[1] Hayes AF. *Introduction to Mediation, Moderation, and Conditional Process Analysis: A Regression-based Approach (3rd Edition)*. New York: The Guilford Press (2022).
